# Case Report: A case of ALS type 6 associated with a *FUS* gene variant and right limb muscle weakness and atrophy as the initial symptom

**DOI:** 10.3389/fgene.2025.1578249

**Published:** 2025-06-18

**Authors:** Xiuping Zhan, Tingting Xuan, Xiaoyan Chen, Jianhang He, Yazhou Ren, Yue Meng, Guisheng Chen, Haining Li

**Affiliations:** ^1^ Department of Neurology, General Hospital of Ningxia Medical University, Yinchuan, China; ^2^ School of Clinical Medicine, Ningxia Medical University, Yinchuan, China; ^3^ Diagnosis and Treatment Engineering Technology Research Center of Nervous System Diseases of Ningxia Hui Autonomous Region, Yinchuan, China

**Keywords:** amyotrophic lateral sclerosis, fused in sarcoma, muscle weakness, muscle atrophy, initial symptom, genetic mutation

## Abstract

Amyotrophic lateral sclerosis (ALS) is a fatal neurodegenerative disease characterized by the progressive degeneration of upper and lower motor neurons. This degeneration results in increasing muscle weakness, ultimately culminating in respiratory failure and death. Mutations in the fused in sarcoma (*FUS*) gene have been identified as a significant cause of ALS. Here, we present the case of a 40-year-old woman who exhibited right limb muscle weakness and atrophy as her initial symptom. Whole genome sequencing revealed a mutation in the *FUS* gene, specifically c.1450_1456delinsCCC (p.Tyr484Profs*44), leading to a diagnosis of ALS type 6 (ALS6). The c.1450_1456delinsCCC (p.Tyr484Profs*44) mutation is a frameshift mutation resulting from a non-triplet base deletion in the coding region of the *FUS* gene. This mutation is novel and has not been previously reported in China or internationally. Furthermore, the onset of muscle weakness and atrophy exclusively in the ipsilateral limb is very rare among ALS patients, and we have found no related reports. This case report aims to enhance medical professionals’ understanding of the complexities associated with ALS caused by *FUS* gene mutations and the onset of ALS symptoms, thereby facilitating more accurate clinical diagnosis and treatment.

## Introduction

ALS is a progressive and fatal neurodegenerative disease characterized by the degeneration of motor neurons in the brain and spinal cord. It is the most prevalent form of motor neuron disease (Grad et al., 2017). The primary clinical manifestations include progressive skeletal muscle weakness, muscle atrophy, fasciculations, bulbar palsy, and signs of pyramidal tract involvement (Yuan et al., 2024). Approximately 90% of patients are diagnosed with sporadic ALS (sALS), while the remaining 10% are diagnosed with familial ALS (fALS) (Riva et al., 2016). Currently, the pathogenesis of ALS remains unclear, with the prevailing belief that its onset results from the interaction between genetic and environmental factors. To date, more than 30 genes have been implicated in the pathogenesis of ALS, with the most commonly identified genes being chromosome nine open reading frame 72 (*C9orf72*), superoxide dismutase 1 (*SOD1*), and *FUS* (van Es et al., 2017). *FUS* is a ubiquitously expressed RNA-binding protein that was first identified as a causative factor in ALS in 2009 (Vance et al., 2009). More than 50 distinct mutations in the *FUS* gene have been identified in patients with ALS, accounting for approximately 4% of fALS cases and less than 2% of sALS cases (Yang et al., 2023). However, the mutation site c.1450_1456delinsCCC (p.Tyr484Profs*44) has not been previously reported. In this report, we present a case of a 40-year-old Chinese female patient with ALS6 associated with this novel variant. This case aims to enhance clinicians’ understanding of ALS resulting from *FUS* gene mutations and to deepen their comprehension of the complexities associated with the onset of ALS symptoms.

## Case presentation

**FIGURE 1:**
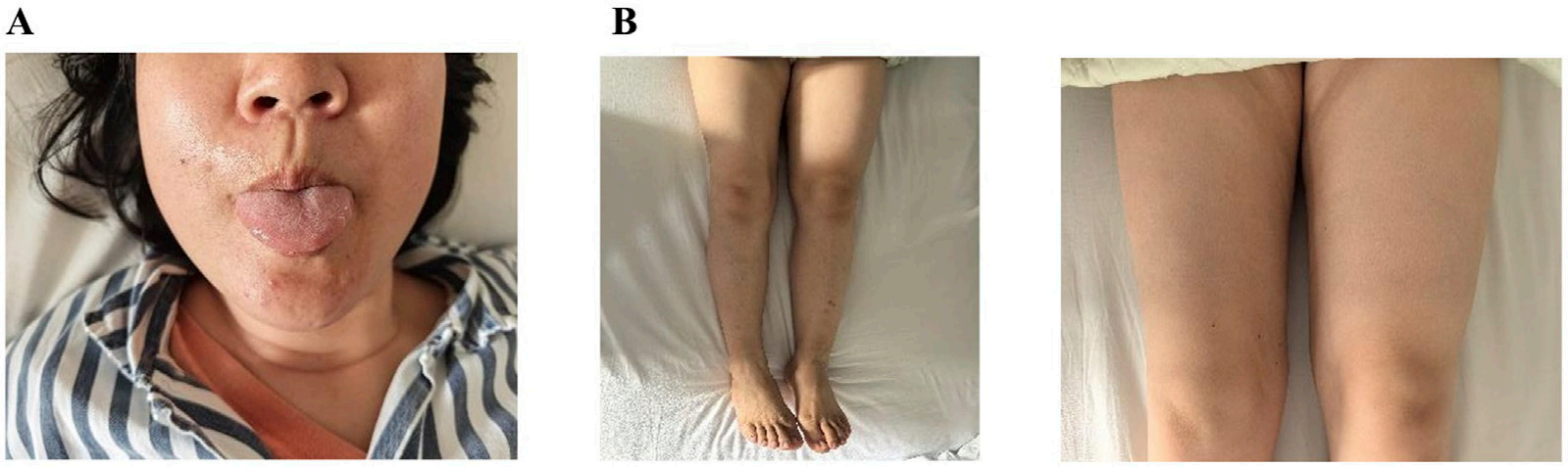
**(A)** Tongue muscle atrophy. **(B)** Quadriceps atrophy: The circumference of the patient’s left thigh was measured at 44.5cm, while the right thigh circumference was 40cm, resulting in a difference of 4.5 cm between the two legs.

**FIGURE 2:**
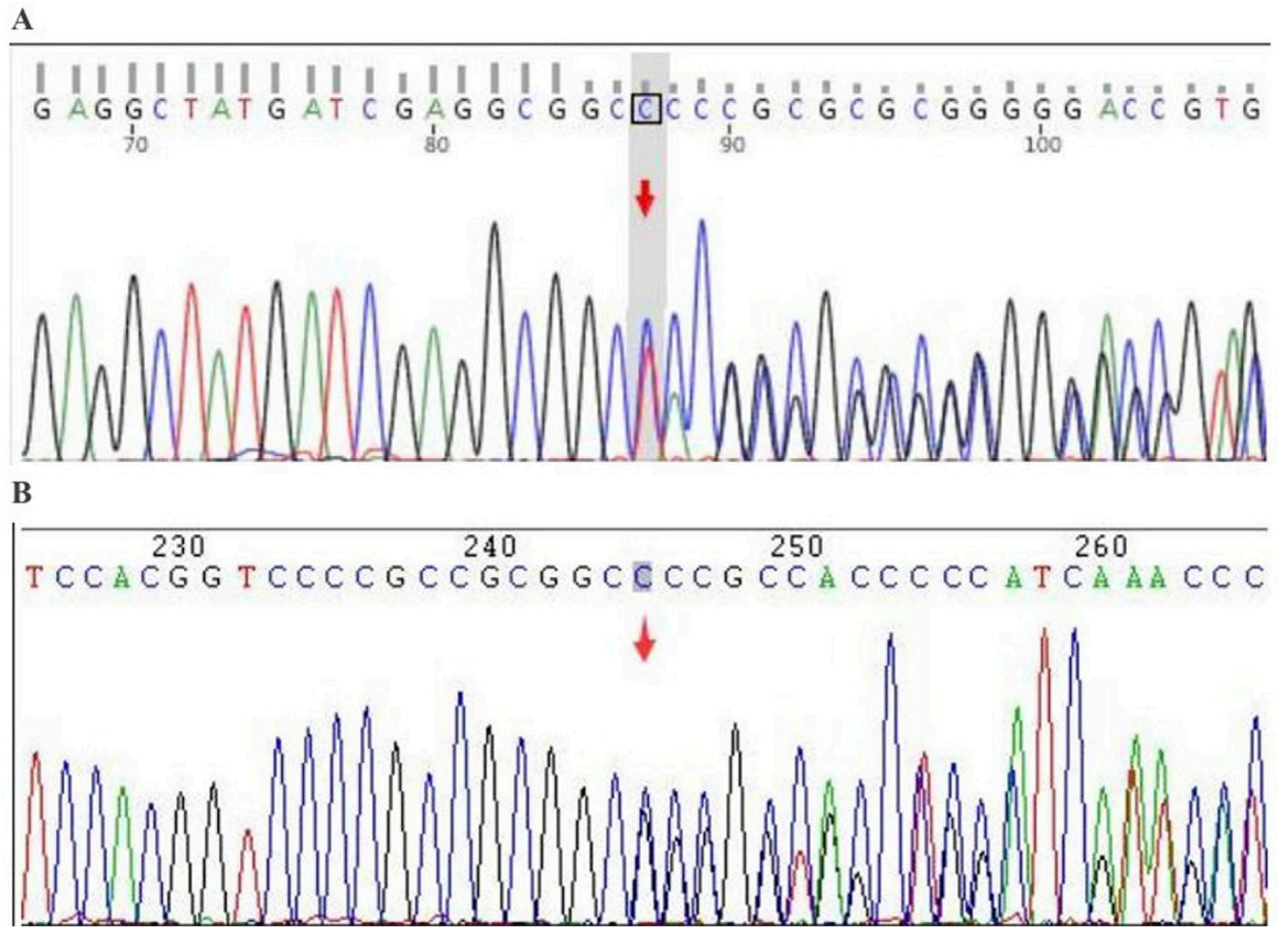
Sanger verification results. Gene: *FUS*; chromosomal location: chr16:31202340_31202345; variant information: c.1450_1455delinsC. **(A)** Forward sequencing (heterozygous deletion-insertion): deletion of TACCGGG and insertion of CCC; **(B)** Reverse sequencing (heterozygous deletion-insertion): deletion of CCCGGTA and insertion of GGG.

In September 2024, a 40-year-old Chinese woman presented to our hospital with right-sided limb muscle weakness and atrophy. She had experienced this weakness 6 months prior to admission, during which she was able to walk independently, and it did not significantly impact her daily activities. Despite multiple visits to local hospitals and undergoing brain and thoracic magnetic resonance imaging (MRI) examinations that revealed no obvious abnormalities, cervical MRI indicated cervical disc herniation. The specific diagnosis and treatment provided by the local hospitals were unclear, and the patient’s symptoms did not improve following their interventions. Consequently, she sought further diagnosis and treatment at Xuanwu Hospital of Capital Medical University in Beijing in April 2024. A brain Computed tomography scan showed no significant abnormalities, while electromyography (EMG) revealed neurogenic damage in the right lower limb and a slowed sensory conduction velocity in the right median nerve. Due to her failure to return for a follow-up visit, the diagnosis remained uncertain. Over the next 5 months, the patient’s right-sided limb weakness progressively worsened, accompanied by atrophy of the tongue muscles and some muscles in her right limbs, prompting her return to our hospital for treatment. Notably, her father had exhibited similar symptoms, characterized by unilateral limb weakness and muscle atrophy, and he passed away at the age of 40. Upon admission, the patient was assessed for atrophy in the tongue muscle and the right biceps, triceps, and quadriceps (Figure 1). The proximal muscle strength of the right upper limb was graded at 2, while the distal muscle strength was graded at 3. The right hand grip strength was measured at 5-, the proximal muscle strength of the right lower limb was graded at 4, the distal muscle strength at 2, and the right foot dorsiflexion at 4. The tendon reflexes in the right limbs were absent, the abdominal reflex was diminished, the right Hoffman and Babinski signs were positive, and the right limb coordination was uncooperative; no significant abnormalities were noted in the remainder of the neurological examination. Additionally, we measured the patient’s left thigh circumference at 44.5 cm and the right thigh circumference at 40cm, resulting in a 4.5 cm difference in leg circumference. The patient underwent testing for serum creatine kinase, lactate levels, rheumatoid and immunological markers, tumor markers, and MRI scans of the brain and cervical spine, all of which revealed no significant abnormalities. Routine cerebrospinal fluid analysis indicated normal pressure, cell count, and glucose levels, with slightly elevated protein. EMG demonstrated extensive neurogenic damage affecting the upper and lower limbs, thoracic paraspinal muscles, and sternocleidomastoid muscles (Table 1). Subsequently, we conducted whole genome sequencing, which identified a c.1450_1456delinsCCC (p.Tyr484Profs*44) mutation in the *FUS* gene. Furthermore, we validated this result through Sanger sequencing (Figure 2). The specific methods are as follows: Blood samples were collected from the patient, and genomic DNA was extracted with QIAamp DNA Blood Mini kit (Qiagen, Hilden, Germany) following the manufacturer’s protocol. (1) Next-generation sequencing: Following the manufacturer’s recommended protocol, the extracted DNA were used as input material for library preparation using the Illumina PCR-Free Prep Kit (Illumina, San Diego, United States). In brief, DNA was fragmented by enzyme and fragmented DNA was end-repaired and A-tailed and indexed adapters added by ligation. The libraries after purification were sequenced on the NovaSeq 6,000 sequencer (Illumina, San Diego, United States). (2) Data analysis: All reads were aligned to the reference human genome (UCSC hg19) by Burrows-Wheeler Aligner. Local realignment and base quality recalibration of the Burrows-Wheeler aligned reads were then performed using the GATK IndelRealigner and GATK BaseRecalibrator, respectively. SNVs and small indels were identified by the GATK UnifiedGenotyper. CNVs and structural variants were analyzed and variants were annotated with in-house pipeline and platform Kingmed Smart Genetic Analyzer. Variants associated with the patient’ phenotype were selected and interpreted. (3) Sanger sequencing: The candidate variants were confirmed in probands and the relatives using PCR and the products were subjected to direct sequencing on 3500XL Genetic Analyzer (Applied Biosystems, Foster City, United States) according to the manufacturer’s instructions. Primers for PCR were designed on primer-blast.

**TABLE 1:** Comparison of the two electromyogram results of the patient.

Testing date Testing items	23 April 2024	6 September 2024
Motor nerve conduction	The latency, amplitude, and conduction velocity of the right median nerve, ulnar nerve, common peroneal nerve, and tibial nerve are within normal ranges	• The amplitudes of the right median, ulnar, radial, musculocutaneous, axillary, suprascapular, and accessory nerves are significantly reduced, whereas the latency and conduction velocity remain within normal limits • The latency, amplitude, and conduction velocity of the right common peroneal and tibial nerves are within normal ranges
Sensory nerve conduction	• The conduction velocity of the right median nerve is decreased, whereas the latency and amplitude remain within normal limits • The latency, amplitude, and conduction velocity of the right ulnar nerve, superficial peroneal nerve, and gastrocnemius nerve are within normal ranges	The latency, amplitude, and conduction velocity of the right median nerve, ulnar nerve, superficial radial nerve, common peroneal nerve, and gastrocnemius nerve are within normal ranges
F wave	The latency and amplitude of the right median and tibial nerves are both normal, with an F wave occurrence rate of 100%	• The latency and amplitude of the right median nerve are both within normal limits, accompanied by an increased F wave repetition rate and an occurrence rate of 85% • The latency and amplitude of the right tibial nerve are within normal ranges, with an F wave occurrence rate of 100%
Needle electrode EMG	The amplitude of the right deltoid muscle motor unit potential is elevated, exhibiting a mixed pattern during maximal voluntary contraction	• A substantial amount of spontaneous electrical activity is detected in the right abductor pollicis brevis while at rest, with weakness evident during light contraction • A considerable amount of spontaneous electrical activity is detected in the right deltoid muscle at rest. During light contraction, the average duration of motor unit potentials extends by 24%, and the voltage increases approximately threefold, demonstrating a pure pattern during maximal contraction • No spontaneous electrical activity is detected in the right sternocleidomastoid muscle at rest. During light contraction, the average duration of motor unit potentials increases by 38%, and the voltage approximately triples, demonstrating a pure pattern during maximal contraction • A substantial amount of spontaneous electrical activity is observed in the paravertebral muscles at the level of the 10th thoracic vertebra while at rest • A considerable amount of spontaneous electrical activity is detected in both the right tibialis anterior and gastrocnemius muscles at rest. During light contraction, the average duration and voltage of motor units remain within normal ranges, while maximal contraction exhibits a pure pattern

**FIGURE 3:**
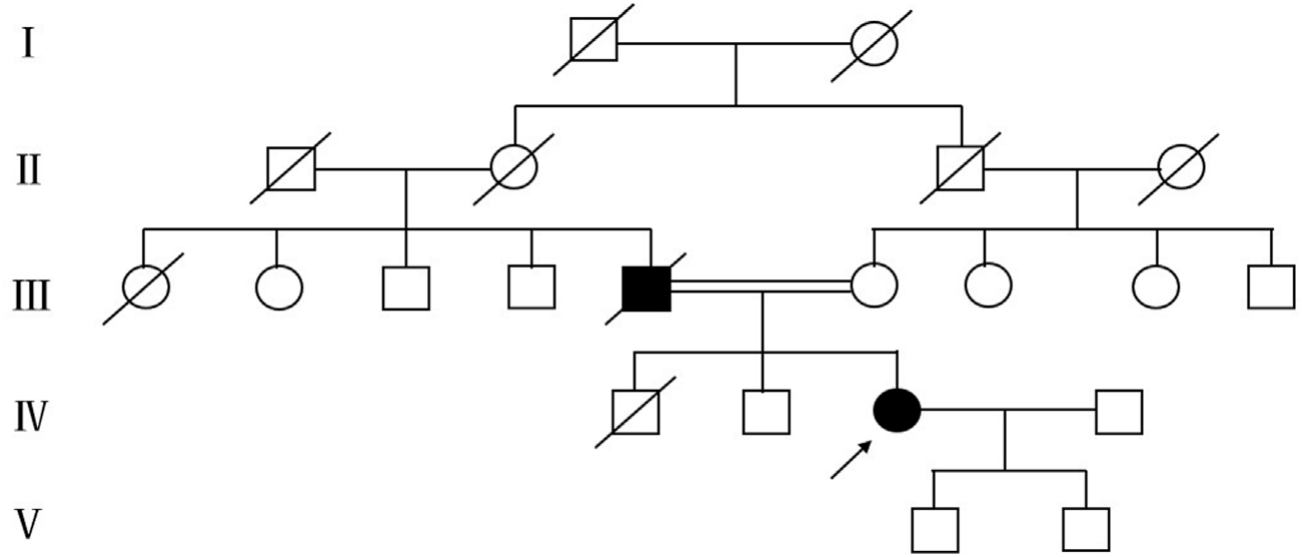
Mutant pedigree map of familial mutations. Circles = females; squares = males; and slashes = deceased.

In summary, the patient exhibits weakness in both the right upper and lower limbs, experiences difficulty with sit-ups, and presents with atrophy in the right biceps brachii, triceps brachii, and quadriceps muscles. Reflexes from the right limb tendon reflexes (including the biceps, triceps, radial, knee, and ankle reflexes) were not elicited. The abdominal reflex is diminished, and the right side shows positive Hoffman and Babinski signs. EMG indicates widespread neurogenic damage, affecting the upper and lower limbs, paravertebral muscles, and sternocleidomastoid muscle. These findings suggest clinical or electrophysiological evidence of upper and lower motor neuron damage in the cervical, thoracic, and lumbosacral spinal regions. According to the revised El Escorial ALS diagnostic criteria, the patient is classified as having clinically definite ALS. Based on genetic testing results, she is ultimately diagnosed with ALS6, and we believe there is a strong possibility that she has fALS. The patient’s father exhibits symptoms similar to hers and has been diagnosed with ALS. The patient’s father has deceased, and the family has not retained his medical records, which renders it impossible to provide his genetic test results. To gain a deeper understanding of the family history, we performed genetic testing on his brother; however, no variants were identified in the whole genome sequencing. A pedigree analysis was subsequently performed (Figure 3). Following the ALS treatment guidelines, we initiated treatment with intravenous edaravone and oral riluzole. After 3 months of follow-up, her symptoms remained relatively stable. Unfortunately, the patient passed away in January 2025, likely due to respiratory failure. This suggests that the progression of ALS is markedly rapid, resulting in a limited survival period.

## Discussion and conclusion

The *FUS* gene, situated on chromosome 16, encodes the FUS protein, a multifunctional RNA-binding protein that plays a crucial role in various RNA metabolic pathways (Reber et al., 2021). Evidence suggests that FUS may assume distinct roles at various stages of development, with its effects closely associated with neural development and neuronal homeostasis (Assoni et al., 2023). Mutations in the *FUS* gene usually lead to changes in the structure and function of the FUS protein. The mutated FUS protein tends to aggregate into pathological aggregates within cells, which are closely related to neuronal cytotoxicity and dysfunction (Aksoy et al., 2020). Currently, two mechanisms have been proposed to elucidate FUS-related neurodegeneration. The first mechanism involves a toxic gain of function, where nuclear FUS accumulates in the cytoplasm and propagates through nerve cells in a prion-like manner. The second mechanism posits that the depletion of FUS from the nucleus may impair transcription, alternative splicing, and DNA repair processes (Abramzon et al., 2020). *FUS* mutations are the second most prevalent genetic alterations in ALS patients in China, particularly among younger individuals. The average age of onset for patients with *FUS* mutations is 39.9 ± 9.5 years (Shen et al., 2024). Mutations in the *FUS* gene are associated with ALS6. The clinical features of ALS6 closely resemble those of the classic ALS phenotype, including muscle weakness, muscle atrophy, and motor neuron dysfunction. The patient discussed in this case primarily exhibited progressive weakness in the right limbs, along with atrophy of the tongue muscles, right biceps, triceps, and quadriceps. Whole genome sequencing revealed a mutation in the *FUS* gene, specifically c.1450_1456delinsCCC (p.Tyr484Profs*44). This frameshift mutation resulted from a non-triplicate base deletion in the coding region of the *FUS* gene, which has not been previously reported. Common mutation sites in *FUS* include p.R521C, p.P525L, p.R521H, p.R495*, p.G504Wfs*12, and p.R521L, with p.R521C being the most frequent mutation among Asian populations and p.P525L being the most prevalent in China (Lu et al., 2022). Unfortunately, while conducting genetic sequencing, we identified mutations in the *FUS* gene but did not perform *C9orf72* repeat expansion analysis. This oversight could introduce significant bias into the research findings and limit their clinical applicability. *C9orf72* repeat expansion is a common genetic cause of ALS, with a high mutation frequency. If not detected simultaneously, co-mutations in *FUS* and *C9orf72* may be overlooked, leading to the erroneous attribution of the disease phenotype solely to the *FUS* mutation. This could result in an overestimation of *FUS*’s pathogenic contribution and an underestimation of *C9orf72*’s population impact, thus distorting genetic epidemiology data. Additionally, *C9orf72* mutations exhibit incomplete penetrance and are strongly associated with familial aggregation of ALS. Failure to detect these mutations may mislead genetic risk assessments, causing family members to miss opportunities for targeted screening and early intervention. Given that *C9orf72* is a key screening gene in ALS research, its omission could raise concerns regarding the methodological rigor of the study and undermine the reliability of the research conclusions. Therefore, in ALS genetic studies, it is essential to perform *C9orf72* repeat expansion analysis alongside the detection of genes such as *FUS* to avoid misjudgments in mechanisms, data bias, and limitations in clinical translational value.

Mutations in the *FUS* gene can result in the loss, alteration, or acquisition of functions of the encoded protein, leading to neurotoxic effects. These mutations can also modify the physical properties of the protein, resulting in the formation of abnormal aggregates that disrupt RNA function and metabolism, protein homeostasis, and axonal cytoskeleton dynamics, ultimately contributing to the development of ALS (Assoni et al., 2023). FUS has been identified as one of the key proteins capable of undergoing liquid-liquid phase separation (LLPS). This process involves the formation of membrane-less liquid droplets, which enable the assembly of higher-order structures critical for the precise spatiotemporal regulation of multiple biological processes. These structures are hypothesized to confer significant advantages in orchestrating dynamic cellular functions, such as transcription and DNA damage repair, by facilitating compartmentalization without membrane boundaries (Naumann et al., 2021; Ishiguro et al., 2021). For several decades, researchers have investigated the unified mechanisms underlying the complex pathogenesis of ALS. Recently, LLPS has been identified as a significant pathway in the development of ALS (Kanekura and Kuroda, 2022; Choi et al., 2023). Numerous proteins, including FUS, are implicated in the pathogenesis of ALS. Mutations in the genes encoding these proteins often promote and accelerate LLPS, eventually resulting in the formation of fibrous aggregates (Choi et al., 2023).

Establishing appropriate animal models of ALS is essential for elucidating its pathogenesis and developing effective intervention strategies. In constructing a mutant *FUS*-edited cell line, researchers utilized advanced gene editing technologies, including the CRISPR-Cas9 system, to precisely modify the endogenous *FUS* gene within the cells. This modification allowed the edited cells to carry gene mutations akin to those observed in ALS patients. Baskoylu et al. (Baskoylu et al., 2022) employed CRISPR-Cas9-mediated genomic editing to create R524S and P525L ALS *FUS* models, aiming to investigate how *FUS* mutations contribute to neuronal dysfunction in individuals with ALS. Dr. Okada’s team employed CRISPR-Cas9 technology to generate *FUS*-ALS mutation in mice possessing non-classical nuclear localization signals H517D (mouse position: H509D) as well as in genome-edited mice (Okada et al., 2024). Their research not only validates the effectiveness of the constructed models but also reveals that the disruption of the nuclear lamina and nuclear pore proteins plays a critical role in the pathological mechanisms underlying ALS (Okada et al., 2024). In this disease-related mouse model of ALS-*FUS*, research has demonstrated that ION 363, a non-allele-specific FUS antisense oligonucleotide, effectively silences FUS and reduces the levels of FUS protein in the brain and spinal cord postnatally, thereby delaying the degeneration of motor neurons (Korobeynikov et al., 2022). These research findings have opened new avenues for the study of ALS and provide a foundation for further investigation. Firstly, these animal models can be utilized to thoroughly explore the changes in downstream signaling pathways induced by *FUS* mutations, clarifying the specific roles of various mutation sites in the disease process. Secondly, inspired by ION 363, further development and testing of novel therapeutic agents can be conducted based on these animal models to identify safer, more effective, and specific intervention strategies. Moreover, ALS animal models with *FUS* mutations can be integrated with models of other pathogenic factors to simulate a more complex disease environment, facilitating a comprehensive analysis of ALS pathogenesis influenced by multiple factors. This holistic approach will ultimately provide a robust theoretical and practical foundation for addressing this challenging disease.

ALS can be categorized into three subtypes based on the sites of onset: limb-onset, bulbar-onset, and respiratory-onset. The majority of patients exhibit limb-onset ALS, characterized by limb weakness, muscle atrophy, muscle fasciculations, and other associated symptoms. A subset of patients presents with bulbar-onset ALS, which is marked by symptoms of bulbar involvement, including dysphagia, slurred speech, and atrophy and fibrillation of the tongue muscles. A small proportion of patients initially present with apnea or dyspnea, often with few or no bulbar and limb symptoms, and are classified as having respiratory-onset ALS (Shen et al., 2024). We summarized the initial symptoms of ALS associated with *FUS* gene mutations (Table 2), with the limb-onset type being the most prevalent. Muscle weakness typically begins in discrete regions of the body and progressively worsens over time and across various areas. It often initiates in one of three primary regions: the face, arms, or legs (Grad et al., 2017). The patient reported in this case exhibited right limb weakness and muscle atrophy as initial symptoms. Although the simultaneous onset of upper and lower limb symptoms on the same side is rare in ALS patients, it aligns with the pathogenesis associated with *FUS* gene variant-related ALS. This case underscores the importance for clinicians to consider genetic mutations when encountering atypical symptoms, enabling timely genetic testing and the formulation of appropriate treatment plans. The brainstem-onset and respiratory-onset forms of ALS are exceedingly rare. During the patient’s hospitalization at our institution, no symptoms indicative of brainstem or respiratory muscle involvement, such as dysphagia, dysarthria, or dyspnea, were observed. In the evaluation of ALS disease, the amyotrophic lateral sclerosis functional rating scale-revised (ALSFRS-R) scale has irreplaceable clinical value. The ALSFRS-R effectively evaluates bulbar, motor, and respiratory functions that are significantly associated with various phenotypes of ALS. This tool not only assesses a patient’s disease status but also predicts their survival time. A judicious clinical application of the ALSFRS-R will enable clinicians to gain a holistic understanding of the patient’s condition and provide a scientific foundation for patient management and prognostic assessments. Despite its subjective dependencies and limitations, this assessment method remains an essential tool for guiding individualized treatment.

**TABLE 2:** Mutations in the *FUS* gene lead to different initial symptoms of ALS diseas**e**.

Initial symptoms	age of onset	Gender	gene	mutation	References
Weakness of the neck flexor muscles	31 years old	male	*FUS*	p.R520C	Lu et al. (2022)
Neck weakness and dysphagia	51 years old	male	*FUS*	p.G466VfsX14	Wang et al. (2020)
Weakness in the right upper limb	60 years old	male	*FUS*	p.G409del	Xiao et al. (2024)
Weakness in the right leg	60 years old	male	*FUS*	p.G507D	Martinelli et al. (2022)
	14 years old	male	*FUS*	p.Q519IfsX527	Picher-Martel et al. (2020)
Weakness in the right foot	17 years old	male	*FUS*	p.P525L	Bodur et al. (2021)
Weakness in the left hand	45 years old	female	*FUS*	p.R495Efs*33	Zou et al. (2021)
Weakness in the left lower limb	33 years old	male	*FUS*	p.R521C	Zou et al. (2021)
Weakness in both hands	19 years old	male	*FUS*	p.P525L	Goldstein et al. (2022)
Weakness in both legs	66 years old	male	*FUS*	p.Q140R	Zou et al. (2021)
Action tremor	18 years old	male	*FUS*	p.P525L	Goldstein et al. (2022)
Involuntary movements of the limbs	43 years old	female	*FUS*	p.R521G	Flies and Veldink (2020)
Dysphonia and diplopia	21 years old	female	*FUS*	p.P525L	Leblond et al. (2016)
Dysphagia and dysarthria	28 years old	female	*FUS*	p.R495X	Calvo et al. (2014)
	19 years old	female	*FUS*	p.P525L	Eura et al. (2019)
Hyperhidrosis	24 years old	female	*FUS*	p.P525L	Chen et al. (2024)

Abbreviations: ALS, amyotrophic lateral sclerosis; *FUS*, fused in sarcoma; ALS6, ALS, type 6; sALS, sporadic ALS; fALS, familial ALS; *C9orf72*, chromosome nine open reading frame 72; *SOD1*, superoxide dismutase 1; MRI, magnetic resonance imaging; EMG, electromyography; LLPS, liquid-liquid phase separation; ALSFRS-R, amyotrophic lateral sclerosis functional rating scale-revised; FVC, forced vital capacity.

ALS must be distinguished from hereditary muscle diseases and mitochondrial disorders during diagnosis. It is characterized by progressive, combined damage to both upper and lower motor neurons, presenting as muscle weakness and atrophy (due to lower motor neuron involvement) alongside increased muscle tone, hyperreflexia, and positive pathological signs (upper motor neuron involvement). In advanced stages, ALS may affect the brainstem, leading to dysphagia and dysarthria. Serum creatine kinase levels are typically mildly elevated or normal, electromyography reveals widespread neurogenic damage, and muscle biopsy shows neurogenic group atrophy. Imaging may occasionally reveal cortical motor atrophy. The prognosis for ALS is poor. Hereditary muscle diseases (e.g., muscular dystrophy) primarily involve myogenic damage, manifesting as symmetric muscle weakness, limited movement, and often accompanied by cardiomyopathy or joint contractures. Onset typically occurs during adolescence, with a clear family history. Creatine kinase is significantly elevated, electromyography shows myogenic changes (low-amplitude, short-duration potentials), muscle biopsy may reveal muscle fiber necrosis, regeneration, and fatty replacement, and genetic testing can identify pathogenic mutations (e.g., DMD, MYOT genes). Mitochondrial diseases are characterized by multi-system involvement. In addition to muscle weakness, they often present with encephalopathy, epilepsy, ophthalmoplegia, exercise intolerance, and metabolic disorders (elevated lactate levels at rest or after exercise). Developmental delay or sensorineural hearing loss may also be present. Muscle biopsy may show fragmented red fibers and cytochrome C oxidase deficiency, while brain MRI may reveal basal ganglia calcifications or stroke-like lesions. The genetic pattern is typically maternal (mitochondrial DNA mutations) or associated with nuclear gene mutations (e.g., POLG). Differentiating these three conditions requires comprehensive analysis of clinical symptoms, electrophysiological features, metabolic markers (lactate, creatine kinase), imaging, and genetic testing. Muscle biopsy and mitochondrial function testing were recommended for the patient in this case to assist with diagnosis; however, she declined due to concerns regarding invasiveness and cost. Based on the comprehensive analysis of the patient’s clinical presentation, electrophysiological features, metabolic markers, imaging, and genetic testing, she was definitively diagnosed with ALS6.

This article presents a case of ALS6 associated with *FUS* gene variants, where right limb muscle weakness and atrophy was the initial symptom. Notably, the specific mutation in the *FUS* gene identified in this patient has not been previously reported in the literature. This finding offers a new perspective on the role of the *FUS* gene in the pathogenesis of ALS. However, the underlying mechanisms remain incompletely understood, necessitating further research to explore these potential pathways. Additionally, the onset characteristics of ALS exhibit significant diversity and individual variation, with symptoms such as muscle weakness and atrophy manifesting in various forms. Therefore, in clinical practice, healthcare providers should remain vigilant for patients who initially present with right limb weakness and carefully assess the possibility of ALS. This observation not only highlights the complexity of ALS onset symptoms but also underscores the need to consider multiple diagnostic possibilities to ensure that patients receive timely and appropriate diagnosis and treatment.

## Data Availability

The datasets presented in this article are not readily available because of ethical and privacy restrictions. Requests to access the datasets should be directed to the corresponding author.
